# Some Practical Notes

**Published:** 1910-04-23

**Authors:** 


### Motoring Notes.

### SOME PRACTICAL NOTES.

If your oil lamps go out frequently when running, it may be owing to the draught-holes in the top being choked up by heavy deposits of carbon.

If a motorist is willing to take a little trouble he can very greatly reduce the rate of wear of his trembler points by periodically reversing the direction of current flow through the system.

Valve springs, like other parts of a motor, will not remain the same for ever. After all methods have been tried and the motor still lacks its usual power, a new set of springs will usually remedy the trouble.

Any small leak which appears in connection with the water circulation should be repaired as promptly as possible, for it quickly grows to a large one, which is much harder to repair. Nothing looks worse than to see a pool of water under a car which has been standing for some time.

To prevent skidding when turning sharp corners check the speed of the car by closing the throttle and applying the brake, if necessary, before reaching the bend; then release the brake and open the throttle a little as the car starts to round the corner, thus making the motor pull the car instead of allowing it to coast round with the brakes on.

Few motorists except those of long experience realise the strain that is put upon the steering mechanism of a car by twisting the wheels around by means of the steering wheel when the vehicle is stationary. Where this is done an unnecessary amount of wear is put on the steering mechanism, and even the steering connections may be bent.

Unless the driver of a motor-car is certain that there is no vehicle close behind him, he should never make a sudden reduction of speed or come to a quick stop without giving a warning signal. The customary way of announcing the intention of slowing or stopping is to raise one hand in the air so that those following may see it readily. Rear-end collisions are undesirable, to say the least, and there are many occasions when they may easily occur if this simple method of signalling is neglected.

Testing a sparking plug on the outside of the cylinder with no compression to influence it is not a correct exposition of its efficiency in producing desired results within the cylinder. The plug may spark beautifully when under observation, but when returned to its proper position no perfect result may be attained. Faulty ignition, if everything connected with the contact-maker, battery, and coil is in order, is usually due to the spark points of the plug being too far apart. The points should be placed as close together as possible without being liable to fouling by small particles of carbon. Closeness of points not only favours production of sparks under compression, but it interposes less resistance to the current passing across the gap and thus makes it less liable to follow some other track.

There is a way of locating a small puncture in an inner tube without the inconvenience of placing the tube in water and getting it wet. This method consists of blowing smoke into the tyre. Deflate the tube completely, and before replacing the valve blow as many mouthfuls of smoke into it as possible; replace the valve and blow up the tube to the required pressure with a pump, and the smoke will issue from any leaks therein.

The cutting of motor tyres by the rims is generally caused either by overloading or lack of sufficient inflation. If the tyres are called upon to carry a greater load than their dimensions are calculated to bear, no amount of inflation will keep them from flattening under the excessive load. This invariably results in the cracking and breaking down of the cover at its weakest point—where the flange engages the beaded edge. Rusty rims are also to be avoided, and they should be occasionally gone over and cleaned of any rust that may have accumulated. A coat or two of enamel will often prevent further corrosion, or the rims may be given a coat of wax. This is a satisfactory way of treating the rusty rims of an old car. The metal should first be well scraped and sandpapered. The wax should then be heated and applied in a liquid state. The wax will not injure the rubber, and by keeping out the air prevents further rusting of the metal. The surface of the rim which comes in contact with the inner tube should be smooth, since, if rough, it is likely to wear and damage the tube. If it is found impossible to get it smooth, the rim should be wrapped with a layer or two of tape, the loose ends being solutioned in place.

Many drivers resort to the slipping of the clutch to enable the car to surmount a hill rather than change to a lower gear. There are many who advocate such a method of driving, while on the other hand there are many who deprecate it. Broadly speaking, it may be said that the practice is a bad one on a long hill when the engine begins to flag early, but if it will obviate changing for just the last few yards over the crest of a rise there is nothing against the practice, except the slight extra wear on the clutch, which can be ignored when the clutch is properly designed and made. However, as some are not acquainted with this method of driving, I give the *modus operandi* of surmounting a hill by slipping the clutch.

The object of the change-speed gear, of course, is to maintain as near as possible an equal engine speed, but it will be easily seen that when one is driving up a hill and the engine begins to lag the clutch may momentarily be withdrawn, the engine allowed to pick up its speed, and the clutch gently let in again, when the momentum will be kept on the car for a short distance. Then the engine will begin to flag again, necessitating another brief release from the heavier portion of its work, after which it will again pick up the car. As a matter of fact one has to keep one's toe on the clutch-pedal all the time, and be prepared to let the engine pick up through exerting some of its power in the propulsion of the car while the clutch is slipping.

